# Changes in fecal microbiota composition and the cytokine expression profile in school-aged children with depression: A case-control study

**DOI:** 10.3389/fimmu.2022.964910

**Published:** 2022-08-19

**Authors:** Zongxin Ling, Yiwen Cheng, Feng Chen, Xiumei Yan, Xia Liu, Li Shao, Guolin Jin, Dajin Zhou, Guizhen Jiang, He Li, Longyou Zhao, Qinghai Song

**Affiliations:** ^1^ Collaborative Innovation Center for Diagnosis and Treatment of Infectious Diseases, State Key Laboratory for Diagnosis and Treatment of Infectious Diseases, National Clinical Research Center for Infectious Diseases, The First Affiliated Hospital, School of Medicine, Zhejiang University, Hangzhou, China; ^2^ Jinan Microecological Biomedicine Shandong Laboratory, Jinan, China; ^3^ Department of Laboratory Medicine, Lishui Second People’s Hospital, Lishui, China; ^4^ Department of Intensive Care Unit, the First Affiliated Hospital, School of Medicine, Zhejiang University, Hangzhou, China; ^5^ Institute of Hepatology and Metabolic Diseases, Hangzhou Normal University, Hangzhou, China; ^6^ Institute of Translational Medicine, The Affiliated Hospital of Hangzhou Normal University, Hangzhou, China; ^7^ Department of Psychiatry, Lishui Second People’s Hospital, Lishui, China

**Keywords:** children, depression, dysbiosis, microbiota-targeted diagnosis, inflammation

## Abstract

Depression in childhood negatively affects the growth and development, school performance, and peer or family relationships of affected children, and may even lead to suicide. Despite this, its etiology and pathophysiology remain largely unknown. Increasing evidence supports that gut microbiota plays a vital role in the development of childhood depression. However, little is known about the underlying mechanisms, as most clinical studies investigating the link between gut microbiota and depression have been undertaken in adult cohorts. In present study, a total of 140 school-aged children (6–12 years) were enrolled, including 92 with depression (male/female: 42/50) and 48 healthy controls (male/female: 22/26) from Lishui, Zhejiang, China. Illumina sequencing of the V3–V4 region of the 16S rRNA gene was used to investigate gut microbiota profiles while Bio-Plex Pro Human Cytokine 27-plex Panel was employed to explore host immune response. We found that, compared with healthy controls, children with depression had greater bacterial richness and altered β-diversity. Pro-inflammatory genera such as *Streptococcus* were enriched in the depression group, whereas anti-inflammatory genera such as *Faecalibacterium* were reduced, as determined by linear discriminant analysis effect size. These changes corresponded to altered bacterial functions, especially the production of immunomodulatory metabolites. We also identified the presence of a complex inflammatory condition in children with depression, characterized by increased levels of pro-inflammatory cytokines such as IL-17 and decreased levels of anti-inflammatory cytokines such as IFN-γ. Correlation analysis demonstrated that the differential cytokine abundance was closely linked to changes in gut microbiota of children with depression. In summary, key functional genera, such as *Streptococcus* and *Faecalibacterium*, alone or in combination, could serve as novel and powerful non-invasive biomarkers to distinguish between children with depression from healthy ones. This study was the first to demonstrate that, in Chinese children with depression, gut microbiota homeostasis is disrupted, concomitant with the activation of a complex pro-inflammatory response. These findings suggest that gut microbiota might play an important role in the pathogenesis of depression in school-aged children, while key functional bacteria in gut may serve as novel targets for non-invasive diagnosis and patient-tailored early precise intervention in children with depression.

## Introduction

Depression in school-aged children, a long-overlooked psychiatric disorder, affects approximately 2.8% of children under the age of 13 and 5.6% of 13–18-year-olds (1). Childhood depression is characterized by sad or irritable mood, decreased self-esteem, sleep disturbance, anhedonia, decreased capacity for fun, social withdrawal or impaired social relationships, and impaired school performance (2, 3). This disorder tends to recur throughout life and is associated with serious complications, including self-harm and suicide. Notably, the prevalence of depression increases markedly after the transition from childhood to adolescence (4). The high prevalence and adverse outcomes have rendered childhood depression a significant public health problem. Additionally, major depressive disorder (MDD) has become a leading risk factor for suicide amongst children. Unlike in adults, depression is difficult to diagnose in children because the symptoms are non-specific and the manifestations may overlap with those normally witnessed at this stage of life. Accordingly, most children with depression go undiagnosed and untreated (2, 5).

Over recent years, childhood depression has received increasing attention from mental health professionals as well as parents. An awareness of the possibility of depression in children can expedite its diagnosis and treatment, thereby avoiding more severe complications later in life. Depression in children can be caused by any combination of factors, including physical health, family history, environment, genetic vulnerability, and biochemical disturbance. Evidence for the heritability of depression in children includes familial transmission; heightened risk for depression in the adult relatives of depressed youths; and estimates of heritability of 40%–80% in studies of twins (2, 6, 7). Environmental factors, together with genetic predisposition, also confer an increased risk for depression (8). A comprehensive meta-analysis of twins indicated that environmental effects account for a 55%-66% risk for major depression (9). Recent preclinical and clinical findings strongly support the existence of a link between gut dysbiosis and depression *via* the microbiota–gut–brain axis. We have previously reported that gut microbiota homeostasis is disrupted in adult patients with MDD, characterized by increased levels of Enterobacteriaceae and *Alistipes* and reduced numbers of *Faecalibacterium*. Notably, we found that *Faecalibacterium* abundance was negatively correlated with the severity of depressive symptoms (10). Interestingly, the transplantation of fecal microbiota derived from patients with depression (“depression microbiota”) into germ-free mice can induce depression-like behavior by altering host metabolism, demonstrating a causal correlation between gut dysbiosis and the development of depression (11). A recent multi-omics-based analysis also revealed that neuroactive metabolites (multiple B vitamins, kynurenic acid, gamma-aminobutyric acid, and short-chain fatty acids) derived from specific depression-associated microbes are involved in the interactions between the gut and the brain and contribute to the pathophysiology of depression (12). Meanwhile, “depression microbiota”-derived molecules and metabolites can promote inflammation in the central nervous system, thereby greatly contributing to the onset of depression (13). Additionally, patients with depression reportedly have higher levels of pro-inflammatory cytokines, acute-phase proteins, chemokines, and cell-adhesion molecules (14, 15). Besides social, psychological, and environmental factors, recent evidence has indicated that the gut microflora, acting through the microbiota–gut–brain axis, may be a key environmental determinant for depression in adults.

Emerging evidence has indicated that the gut microbiota undergoes age-related changes; however, relatively few studies have considered the effects of age on the gut microbiota when exploring the pathogenesis of depression. Moreover, most clinical studies investigating the association between the gut microbiota and this condition have involved adult cohorts. Given the differences in environmental exposures and life trajectories between children and adults, further studies investigating the connection between gut dysbiosis and depression in childhood are urgently needed. Accordingly, the aim of this study was to explore in detail the structure and composition of the fecal microbiota in pediatric patients with MDD from Lishui using high-throughput 16S rRNA gene sequencing on the MiSeq platform, as well as identify putative associations between altered microbial profiles and host cytokine expression levels. Our findings provide novel insights into the etiology of depression in children as well as contribute to non-invasive diagnosis and personalized microbiota-targeted therapy for depression in childhood.

## Methods

### Participants’ enrollment

A total of 92 pediatric patients with newly diagnosed MDD (age 6-12 years) according to the criteria of the Hamilton Depression Scale (HAMD), and/or the Diagnostic and Statistical Manual of Mental Disorders Fifth Edition (DSM-V), and/or the third version of Chinese Classification of Mental Disorder (CCMD-3), were recruited from Lishui, Zhejiang (China) from November 2019 to April 2021, while 48 healthy children with similar age and sex distribution were enrolled as controls. The pediatric MDD patients were enrolled in the outpatient clinics of Lishui Second People’s Hospital (Zhejiang, China) and diagnosed by two experienced pediatric psychiatrists. Meanwhile, their schoolmates were recruited randomly, evaluated systematically, and selected as healthy control. Those schoolmates with HAMD more than 7 would be exclude from healthy controls. Dietary and other socio-demographic information was obtained *via* questionnaires. All these participants lived in the Liandu district of Lishui, with similar birth modes, dietary habits, lifestyles, and environment. These protocols for the study were approved by the Ethics Committee of Lishui Second People’s Hospital and written informed consent was obtained from their guardian before enrollment. The detailed demographic data and medical history were collected using a set of questionnaires (**Table S1**). The exclusion criteria included: age < 6 or > 13 years; body mass index (BMI)  > 28 kg/m^2^; active respiratory or intestinal infections; autism spectrum disorder, anorexia nervosa, bipolar disorder, attention-deficit/hyperactivity disorder, mania; antibiotic, prebiotic, probiotic, or synbiotic administration in the previous month; antidepressant, mood stabilizers or other psychiatric drugs in last 1 months; autoimmune diseases.

### Sample collection and bacterial DNA extraction

The sample collection, processing and banking are according to our previous standardized protocols. Briefly, approximately 2g of a fresh fecal sample was collected in a sterile plastic cup, and stored at -80°C after preparation within 15 min until use. Serum samples from these participants were obtained using their fasting blood in the early morning. Bacterial genomic DNA was extracted from 300 mg of homogenized feces using a DNA Stool Mini Kit (QIAGEN, Hilden, Germany) according to the manufacturer’s instructions (16, 17). The amount of DNA was determined using a NanoDrop ND-1000 spectrophotometer (Thermo Electron Corporation, Boston, MA, USA) and the quality of DNA was checked by agarose gel electrophoresis. All DNA was stored at -20°C before further analysis.

### Amplicon library construction and sequencing

The protocols of amplicon library construction and sequencing were conducted as our previous studies (16–18). The details were shown as follows: amplicon libraries were constructed with Illumina sequencing-compatible and barcode-indexed bacterial PCR primers 341F (5’-CCTACGGGNGGCWGCAG-3’)/785R (5’-ACTACHVGGGTATCTAATCC-3’), which target the V3-V4 regions of the 16S rRNA gene (19). All PCR reactions were performed with KAPA HiFi HotStart ReadyMix using the manufacturer’s protocol (KAPA Biosystems) and approximately 50 ng of extracted DNA per reaction. Thermocycling conditions were set at 95°C for 1 min, 55°C for 1 min, then 72°C for 1 min for 30 cycles, followed by a final extension at 72°C for 5 min. All PCR reactions were performed in 50 μl triplicates and combined after PCR. The amplicon library was prepared using a TruSeq™ DNA sample preparation kit (Illumina Inc, San Diego, CA, USA). Prior to sequencing, the PCR products were extracted with the MiniElute^®^ Gel Extraction Kit (QIAGEN) and quantified on a NanoDrop ND-1000 spectrophotometer (Thermo Electron Corporation) and Qubit 2.0 Fluorometer (Invitrogen). The purified amplicons were then pooled in equimolar concentrations and the final concentration of the library was determined by Qubit (Invitrogen). Negative DNA extraction controls (lysis buffer and kit reagents only) were amplified and sequenced as contamination controls. Sequencing was performed on a MiSeq instrument (Illumina) using a 300 × 2 V3 kit together with PhiX Control V3. MiSeq sequencing and library construction were performed by technical staff at Hangzhou KaiTai Bio-lab.

### Bioinformatic analysis

Based on our previous studies, the 16S rRNA gene sequence data set generated from the Illumina MiSeq platform was inputted to QIIME2 (version 2020.11), and all steps of sequence processing and quality control were performed in QIIME2 with default parameters (16, 17, 20, 21). Before the following data analysis, these reads of each sample were normalized to even sampling depths and annotated using the Greengenes reference database (version 13.8) with both the RDP Classifier and UCLUST version 1.2.22 methods implemented in QIIME2. α-diversity indices, including the observed species, abundance-based coverage estimator (ACE), Chao1 estimator, Shannon, Simpson, Evenness and PD whole tree indices, were calculated at a 97% similarity level. β-diversity was measured by the unweighted UniFrac, weighted UniFrac, jaccard and Bray-Curtis distances calculated by QIIME2, which were visualized by principal coordinate analysis (PCoA). The differences in the composition of the fecal microbiota at different taxonomic levels were analyzed with Statistical Analysis of Metagenomic Profiles (STAMP) software package v2.1.3 and the linear discriminant analysis (LDA) effect size (LEfSe) method. Only bacterial phylotypes with an average relative abundance of more than 0.01% were selected for the LEfSe analysis. Krona chart was plotted using taxonomy summary data obtained from QIIME Krona chart displays abundance and hierarchy simultaneously using a radial space-filling display and features a red-green color gradient, signifying the average BLAST hits e-values within each taxon (22). PiCRUSt v1.0.0 was used to identify predicted gene families and associated pathways from inferred metagenomes of taxa of interest identified from the compositional analyses.

### Multiplex cytokine analysis

Serum cytokines, chemokines and growth factors were probed using Bio-Plex Pro Human Cytokine 27-plex Panel (M50-0KCAF0Y, Bio-Rad, Hercules, CA, USA) multiplex magnetic bead-based antibody detection kits following manufacturer’s instructions. Based on the Luminex^®^ xMAP^®^ technology, the assays are capable of simultaneously quantifying 27 targets including interleukin-1β (IL-1β), IL-1 receptor antagonist (IL-1ra), IL-2, IL-4, IL-5, IL-6, IL-7, IL-8, IL-9, IL-10, IL-12(p70), IL-13, IL-15, IL-17, Eotaxin, Fibroblast growth factor-basic (FGF-basic), granulocyte colony-stimulating factor (G-CSF), granulocyte-macrophages colony-stimulating factor (GM-CSF), interferon gamma (IFN-γ), interferon gamma-inducible protein 10 (IP-10), monocyte chemotactic protein-1 (MCP-1), macrophages inflammatory protein-1α (MIP-1α), platelet-derived growth factor (PDGF-bb), MIP-1β, regulated upon activation normal T-cell expressed and secreted (RANTES), tumor necrosis factor-alpha (TNF-α), and vascular endothelial growth factor (VEGF). The assays were run on the Luminex^®^ 200™ system (Bio-Rad) and fluorescence values were collected. A standard curve was derived using the different concentrations of the assay standards. Data was acquired using the Bio-Plex Array Reader system 2200. The results expressed as picogram per milliliter (pg/mL) using the standard curves integrated into the assay and Bio-Plex Manager v5.0 software with reproducible intra- and inter-assay CV values of 5-8% (16, 17, 23).

### Statistical analysis

White’s nonparametric *t*-test, independent *t*-test, or Mann-Whitney *U*-test were applied for continuous variables. Pearson chi-square or Fisher’s exact test were used for categorical variables between groups, Spearman’s rank correlation test was utilized for correlation analyses. Statistical analysis was performed using the SPSS v19.0 (SPSS Inc., Chicago, IL) and STAMP v2.1.3 (24). False-discovery rate (FDR) was calculated according to Benjamini-Hochberg, FDR-corrected p values were denoted as Q_FDR_ and was used when performing all untargeted screening analyses of different taxa. The predictive power was evaluated by receiver operating characteristics (ROC) and area under the curve (AUC) analysis to determine the ability of the differential bacteria to accurately predict childhood depression. R software ggplot2 and pheatmap packages and GraphPad Prism v6.0 were used for preparation of graphs. All tests of significance were two sided, and p<0.05 or corrected p<0.05 was considered statistically significant.

### Accession number

The sequence data from this study are deposited in the GenBank Sequence Read Archive with the accession number PRJNA846994.

## Results

### Characteristics of patients

A total of 140 school-aged children were included in this study, including 92 recently diagnosed with MDD (male/female: 42/50; age: 8.84 ± 1.89 years) and 48 healthy controls (male/female: 22/26; age: 9.27 ± 2.11 years). No differences in clinical characteristics such as BMI (21.86 ± 2.33 in MDD patients *vs.* 21.33 ± 2.27 in the controls), birth mode (vaginal delivery: 68 for MDD patients *vs.* 37 for the controls; cesarean section: 24 among MDD patients vs. 11 among the controls), and feeding mode (all mixed feeding) were detected between the two groups (p > 0.05). All the children newly diagnosed with MDD were treatment-naive. The average HAMD score for children with MDD was 24.0 ± 4.52, significantly higher than that for the healthy controls (4.2 ± 2.48; p < 0.05). Among the children with MDD, 12 had a family history of a psychiatric disorder such as MDD or schizophrenia.

### Fecal microbiota structure was altered in children with MDD

**Figure 1:**
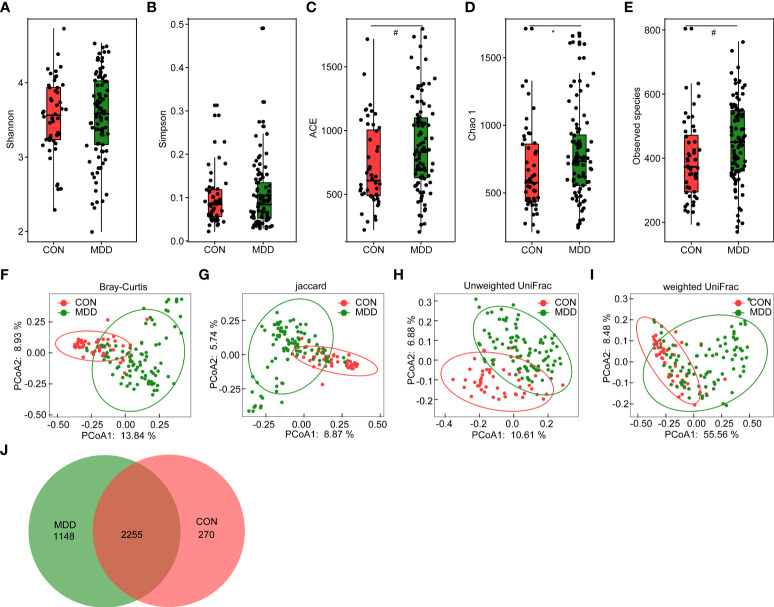
Altered overall structure of the fecal microbiota in patients with childhood major depressive disorders (MDD). The diversity indices of Shannon **(A)** and Simpson **(B)**, and the richness indices of ACE **(C)**, Chao1 **(D)**, and the observed OTUs **(E)** were used to evaluate the overall structure of the fecal microbiota in childhood MDD patients and the healthy controls. The data are presented as mean ± standard deviation. Unpaired *t* tests (two tailed) were used to analyze the variation between the groups. *p < 0.05 and ^#^p < 0.01 compared with the control group. Principal coordinate analysis (PCoA) plots of individual fecal microbiota based on Bray–Curtis **(F)**, jaccard **(G)**, and unweighted **(H)** and weighted **(I)** UniFrac distances in childhood MDD patients and the healthy controls. Each symbol represents a sample. The Venn diagram illustrates the overlap of OTUs in childhood MDD-associated microbiota and healthy controls **(J)**.

For microbiota analyses, we obtained 3,883,979 high-quality reads (1,222,781 for healthy controls and 2,661,198 for children with MDD), with an average of 27,742 reads per sample. In total, we identified 3,673 OTUs (unique bacterial phylotypes) among the fecal microbiota, attaining a Good’s coverage of 98.87%, indicating that most of the fecal bacteria had been detected. The α-diversity of the fecal microbiota defines its richness (number of OTUs) and evenness (relative abundance of the OTUs) either qualitatively (richness and Chao1 indices) or quantitatively (Shannon and Simpson indices). Interestingly, the Shannon and Simpson index values were not significantly different between the healthy controls and the children with MDD (**Figures 1A, B**), whereas the richness index values—ACE, Chao1, and observed OTUs—were significantly higher in children with MDD than in the healthy controls (**Figures 1C–E**). To characterize the global differences between the fecal microbial communities of the two groups, PCoA plots (bacterial β-diversity) were generated based on the Bray–Curtis (*R* ^2^ = 0.079), Jaccard (*R* ^2^ = 0.052), unweighted UniFrac (*R* ^2^ = 0.054), and weighted UniFrac (*R* ^2^ = 0.181) distances. The results showed significant separation between the fecal samples of the pediatric patients with MDD and those of healthy children despite the significant interindividual variation observed (ADONIS test: p < 0.01; **Figures 1F–I**). Additionally, a Venn diagram of the shared OTUs between the children with MDD and healthy children showed that a total of 2,255 OTUs were shared between the two groups, while 1,148 and 270 OTUs were unique to children with MDD and healthy children, respectively (**Figure 1J**).

### Differences in fecal microbiota composition between children with MDD and healthy controls

**Figure 2:**
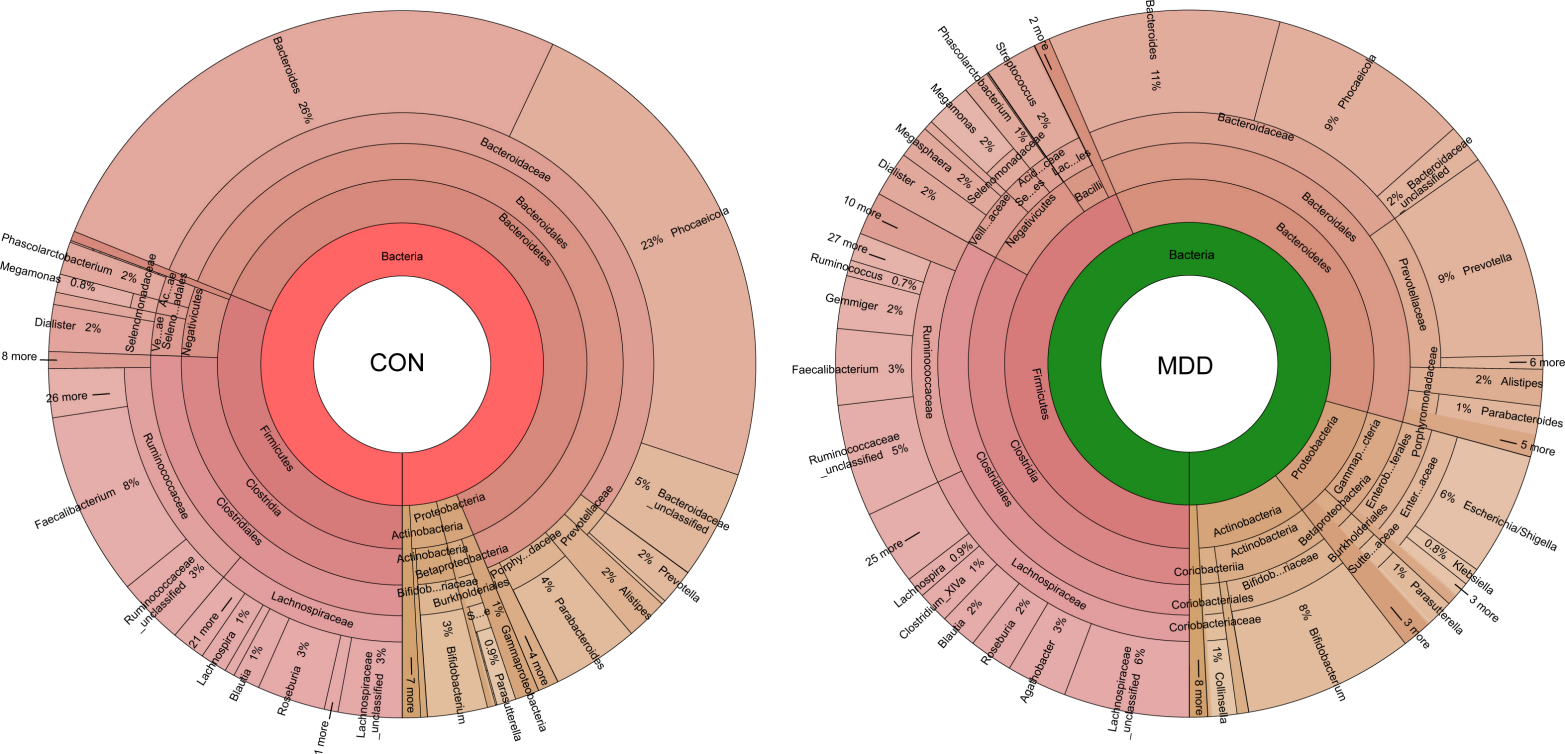
Krona charts showing the taxonomic identification and relative abundance of the most abundant bacterial OTUs recorded in childhood MDD patients and healthy controls. These taxa represent the internal core microbiota at the individual level.

**Figure 3:**
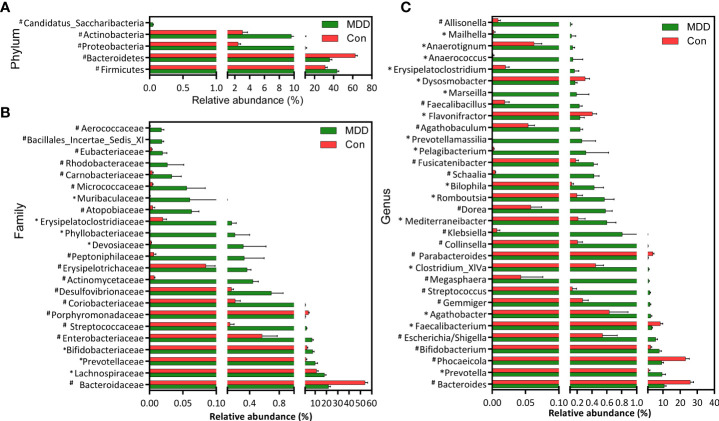
Differential bacterial taxa between childhood MDD patients and the healthy controls. Comparisons of the relative abundance of the abundant bacterial taxa at the level of bacterial phylum **(A)**, family **(B)**, and genus **(C)**. The data are presented as the mean ± standard deviation. Mann–Whitney *U*-tests were used to analyze variation between childhood MDD patients and the healthy controls. *p < 0.05 and ^#^p < 0.01 compared with the control group.

**Figure 4:**
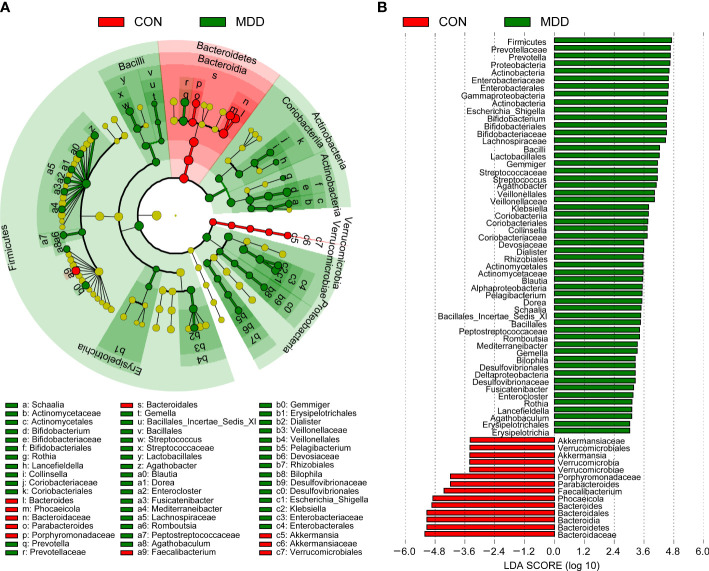
Taxonomic differences of the fecal microbiota between childhood MDD patients and the healthy controls. LEfSe identified the features of the fecal microbiota that are discriminative with respect to childhood depression using the LDA model results for the bacterial hierarchy **(A)**, while LDA coupled with effect size measurements identified the most differentially abundant taxa between the two groups **(B)**. Only the taxa meeting a significant LDA threshold value of > 3 are shown.

Bacterial community composition at different taxonomic levels was compared between patients with MDD and healthy children to identify the drivers of community separation. In total, the sequence reads were classified into 11 phyla, 89 families, and 246 genera in the fecal microbiota of the children using the RDP classifier. Krona radial space-filling charts showed the mean relative abundances of bacterial taxa in children with MDD and healthy children from phylum to genus levels (starting at the inner circle; **Figure 2**). The charts clearly demonstrated that the fecal microbiota of the children was dominated by the phyla Firmicutes, Bacteroidetes, Actinobacteria, and Proteobacteria. **Figure 3** illustrates the differentially abundant bacteria at different taxonomic levels between the pediatric patients with MDD and healthy children. Specifically, regarding the relative abundance of microbiota at the phylum level, the proportion of Firmicutes, Actinobacteria, Proteobacteria, and Candidatus_Saccharibacteria was significantly higher in child patients with MDD while that of Bacteroidetes was lower (p < 0.05, **Figure 3A**). Interestingly, the ratio of Firmicutes to Bacteroidetes (changes in which can be an indicator of gut dysbiosis) was significantly greater in children with MDD than in healthy children (p < 0.05, **Supplementary Figure 1**). At the family level, the proportions of Lachnospiraceae, Prevotellaceae, Bifidobacteriaceae, Enterobacteriaceae, Streptococcaceae, and Coriobacteriaceae were significantly greater, while those of other families, such as Bacteroidaceae and Porphyromonadaceae, were smaller, in patients with MDD than in the healthy controls (p < 0.05, **Figure 3B**). At the genus level, the proportions of 26 genera, such as *Prevotella*, *Bifidobacterium*, *Escherichia*/*Shigella*, *Agathobacter*, *Gemmiger*, *Streptococcus*, *Megasphaera*, *Clostridium*_XlVa, and *Collinsella*, were significantly greater in children with MDD than in the controls, while those of 6 other functional genera—*Bacteroides*, *Phocaeicola*, *Faecalibacterium*, *Parabacteroides*, *Flavonifractor*, and *Dysosmobacter*—were markedly lower (p < 0.05, **Figure 3C**). LEfSe analysis showed that many key functional taxa (biomarkers) were different between the children with MDD and the healthy controls at all taxonomic levels (LDA score > 3, p < 0.05) (**Figure 4**). A representative cladogram of the most differentially abundant taxa between the two cohorts, demonstrating the changes in fecal microbiota composition in child patients with MDD, is shown in **Figure 4A**. At the genus level, *Prevotella* (LDA=4.7, p<0.01), *Escherichia*/*Shigella* (LDA=4.5, p<0.01), *Bifidobacterium* (LDA=4.5, p<0.01), *Streptococcus* (LDA=4.1, p<0.01), *Gemmiger* (LDA=4.1, p<0.01), *Agathobacter* (LDA=4.1, p<0.01), *Klebsiella* (LDA=3.8, p<0.01), and *Collinsella* (LDA=3.7, p<0.01), among others, were biomarkers for the childhood MDD group, while *Bacteroides* (LDA=4.9, p<0.01), *Faecalibacterium* (LDA=4.4, p<0.01), *Parabacteroides* (LDA=4.2, p<0.01) and *Akkermansia* (LDA=3.4, p<0.01) were biomarkers for the healthy control group (**Figure 4B**).

### A fecal microbiota-based signature could discriminate between children with MDD and healthy controls

**Figure 5:**
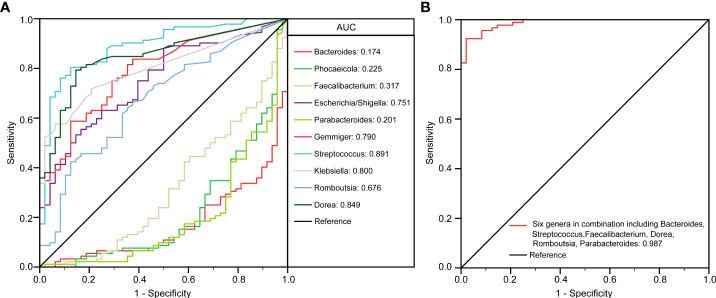
The differential genera as childhood MDD diagnostic markers. Receiver operating characteristic (ROC) curves for the differential genera such as *Bacteroides*, *Phocaeicola*, *Faecalibacterium*, *Escherichia/Shigella*, *Parabacteroides*, *Gemmiger*, *Streptococcus*, *Klebsiella*, *Romboutsia*, *Dorea* alone **(A)** or in combination including *Bacteroides*, *Streptococcus*, *Faecalibacterium*, *Dorea*, *Romboutsia* and *Parabacteroides* **(B)** used to discriminate childhood MDD patients from healthy controls. AUC, the area under the receiver operating characteristic curve.

To identify biomarkers that made a significant contribution to the prediction performance, we performed a receiver operating characteristic (ROC) curve analysis. The results of the LEfSe analysis indicated that several genera could be used as potential biomarkers to discriminate between child patients with MDD and healthy children. Important biomarkers, such as *Bacteroides*, *Phocaeicola*, *Faecalibacterium*, *Escherichia/Shigella*, *Parabacteroides*, *Gemmiger*, *Streptococcus*, *Klebsiella*, *Romboutsia*, and *Dorea*, were assessed for their potential discriminating value. Using only one of the differentially abundant genera as a predictor, we obtained an area under the ROC curve (AUC) ranging from 0.174 to 0.891 (**Figure 5A**). The results indicated that an increased abundance of *Streptococcus* was the best predictor for MDD in children (AUC: 0.891). We further utilized multiple logistic regression analysis to identify the best combinations of the key functional genera that could distinguish child patients with MDD from healthy controls. We found that combinations of six of the above-mentioned genera—*Bacteroides*, *Streptococcus*, *Faecalibacterium*, *Dorea*, *Romboutsia*, and *Parabacteroides*—yielded improved diagnostic performance, relative to each bacterium alone (AUC: 0.987) (**Figure 5B**). Meanwhile, *Phocaeicola*, *Escherichia/Shigella*, *Gemmiger*, and *Klebsiella* were excluded from further analysis because combinations with other key functional genera yielded lower AUC values compared with each bacterium alone.

### General functional profile of the microbiota associated with MDD in childhood

**Figure 6:**
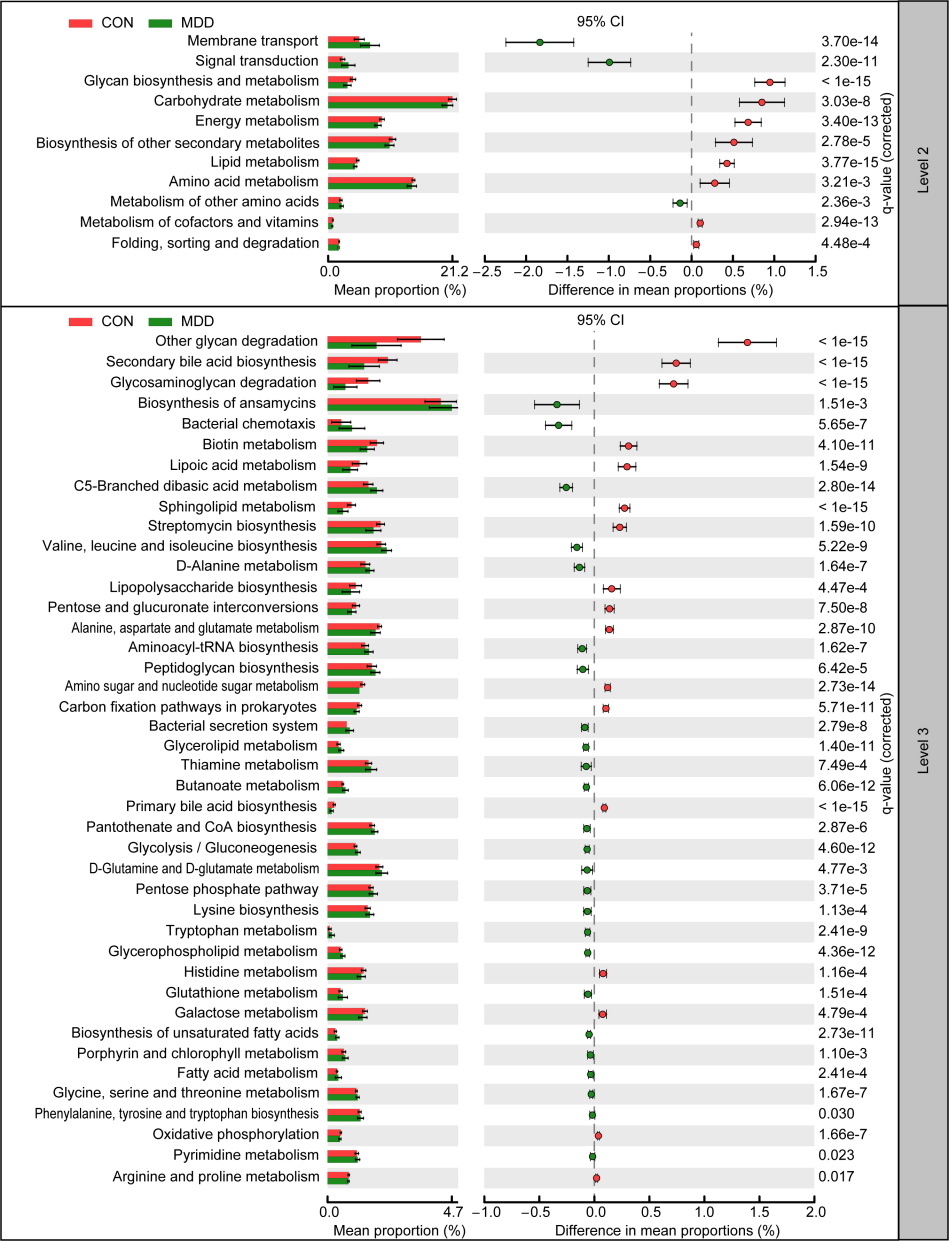
PiCRUSt-based examination of the fecal microbiota of childhood MDD patients and the healthy controls. The different bacterial functions were evaluated between them based on two-sided Welch’s *t*-test. Comparisons between the groups for each KEGG functional category (levels 2 and 3) are shown by percentage. The Benjamini–Hochberg method was used for multiple testing correction based on the false discovery rate (FDR) through STAMP.

The function of the microbiota associated with MDD in children was explored using the PiCRUSt algorithm. This algorithm can predict the abundances of functional categories within the Kyoto Encyclopedia of Genes and Genomes (KEGG) orthology (KO) database based on closed-reference OTU picking, thereby identifying metabolic and functional changes in fecal microbiota. The general functional profile of the childhood MDD-associated fecal microbiota is shown in **Figure 6**. Among the 64 level-2 KEGG pathways, we identified 11 categories displaying marked differential abundance between children with MDD and healthy children (p < 0.05), 3 of which were enriched (membrane transport, signal transduction, and metabolism of other amino acids) and 8 decreased (folding, sorting and degradation, biosynthesis of other secondary metabolites, amino acid metabolism, lipid metabolism, metabolism of cofactors and vitamins, energy metabolism, carbohydrate metabolism, and glycan biosynthesis and metabolism) in the MDD group. At level 3, a total of 42 KEGG pathways were identified as displaying significantly differential activity between the fecal microbiota of the two groups (p < 0.05). Specifically, 25 pathways, including fatty acid metabolism, biosynthesis of unsaturated fatty acids, bacterial secretion system, and lysine biosynthesis, showed higher activity in the childhood MDD-associated fecal microbiota, while 17 pathways, such as lipopolysaccharide biosynthesis, secondary bile acid biosynthesis, glycosaminoglycan degradation, and primary bile acid biosynthesis, showed a prominent reduction in activity. Collectively, our findings suggested that the altered functional potential of the bacterial assemblages in the fecal microbiota associated with MDD in childhood, such as increased fatty acid metabolism and decreased bile acid biosynthesis, may play a role in the pathogenesis and progression of the condition.

### Correlations between differentially abundant genera and host cytokines levels

**Figure 7:**
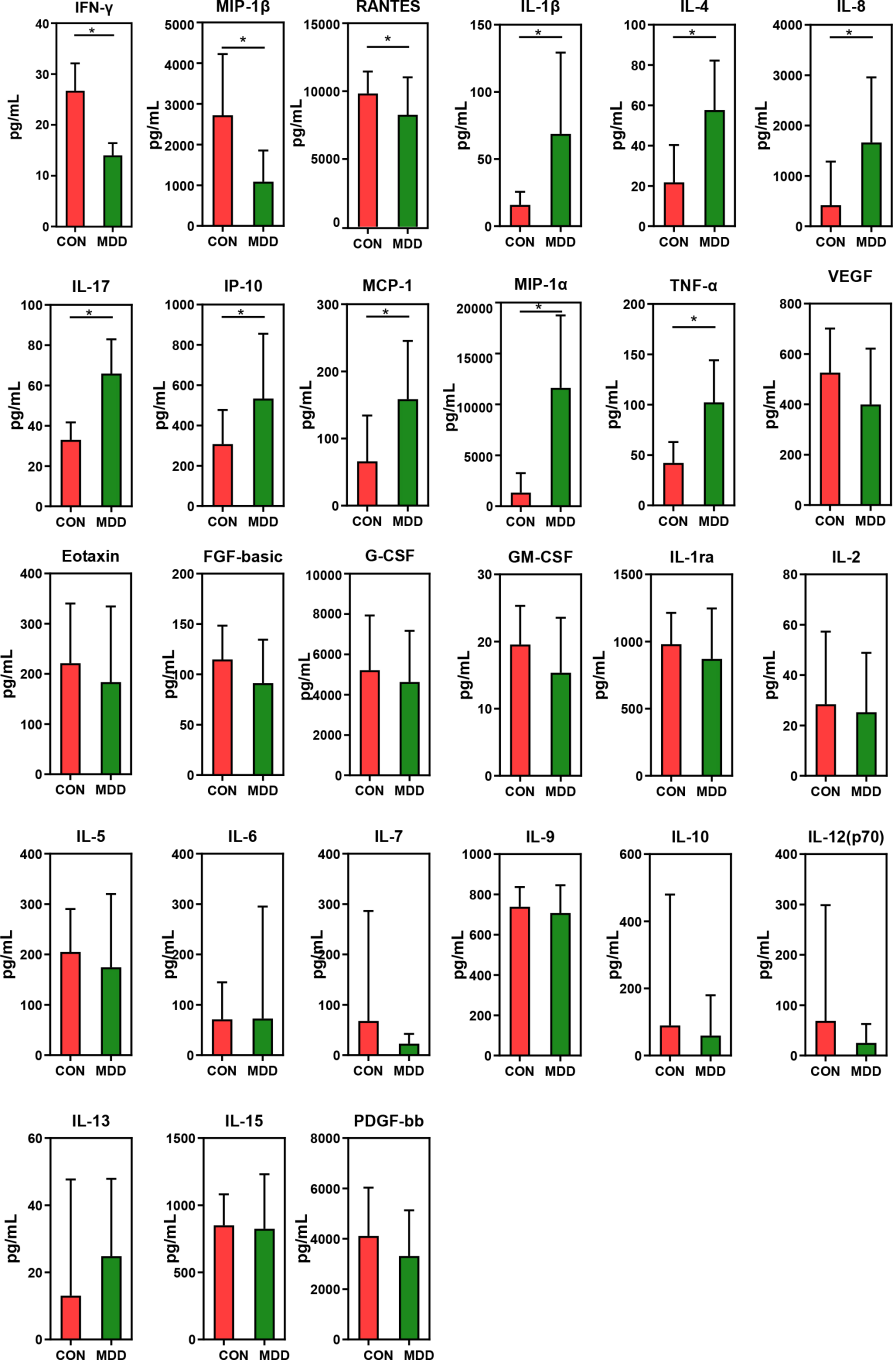
Mean (SEM) concentrations (pg/ml) of 27 pro- and anti-inflammatory cytokines and chemokines in childhood MDD patients and in healthy controls determined using Bio-Plex immunoassays. The concentrations of IL-1β, IL-4, IL-8, IL-17, IP-10, MCP-1, MIP-1α and TNF-α increased significantly in childhood MDD patients, while those of IFN-γ, MIP-1β and RANTES decreased significantly. *p < 0.05.

**Figure 8:**
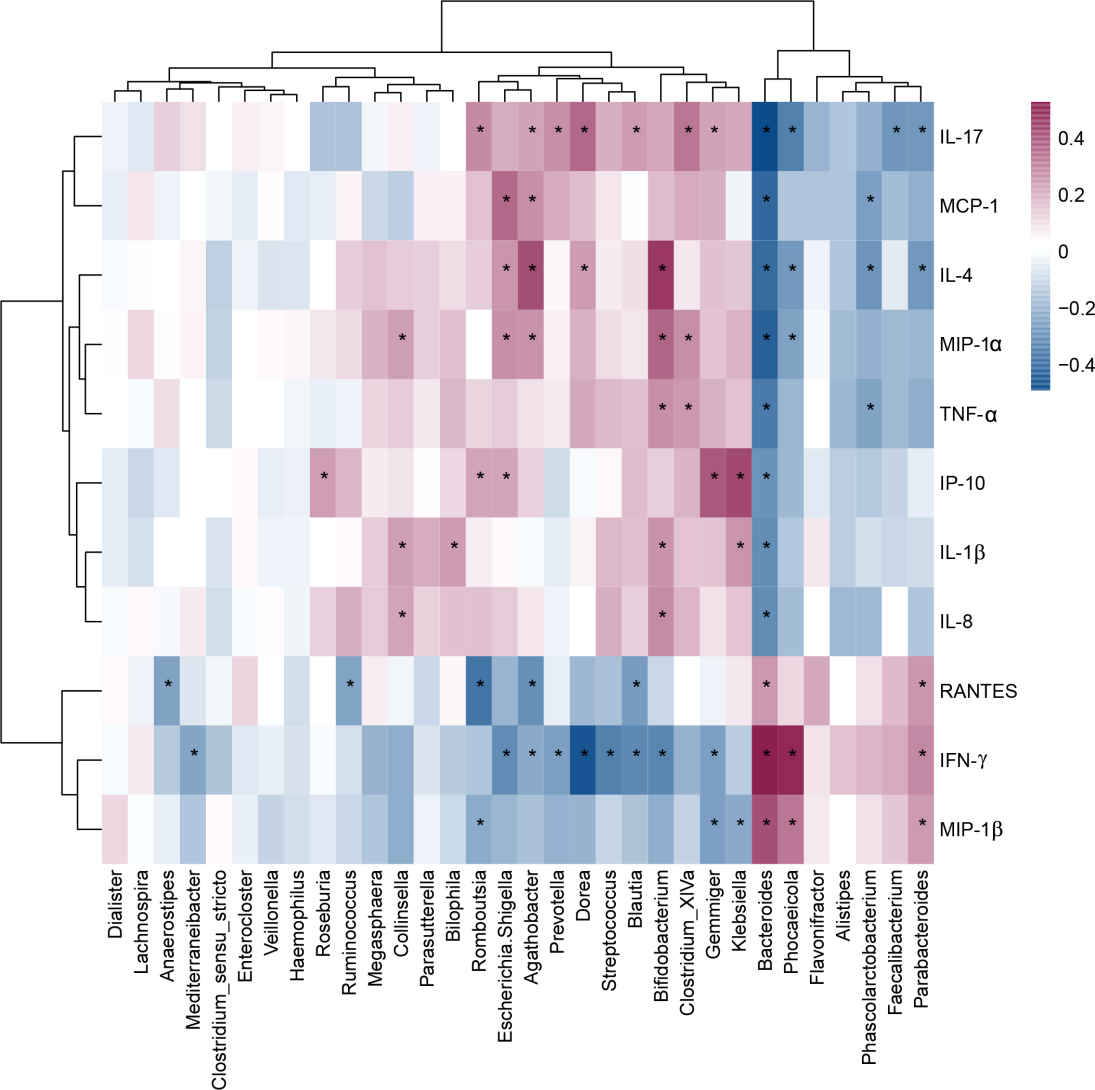
Correlation between fecal microbiota, and pro- and anti-inflammatory cytokines and chemokines in childhood depression. The heatmap shown Pearson’s correlation coefficients between differential genera and host immunity in childhood MDD patients. Pearson correlation (r) and probability (p) were used to evaluate statistical importance. *p < 0.05.

As shown in **Figure 7**, children with MDD exhibited complex changes in cytokine expression levels. Of the 27 cytokines examined, 8 (IL-1β, IL-4, IL-8, IL-17, IP-10, MCP-1, MIP-1α, and TNF-α) were noticeably upregulated in child patients with MDD relative to healthy children while 3 (IFN-γ, MIP-1, and RANTES) were markedly downregulated (all p < 0.05). Next, to assess whether there was a reciprocal relationship between altered host immunity and the key functional bacteria in the child patients with MDD, we performed a Pearson’s correlation analysis. Heatmaps were created based on Pearson’s correlation coefficient (r, **Figure 8**). Interestingly, in children with MDD, the key functional genus, *Bacteroides*, was negatively associated with the above-mentioned upregulated cytokines and positively correlated with the downregulated cytokines. The genera displaying increased abundance, such as *Prevotella*, *Bifidobacterium*, *Escherichia*/*Shigella*, *Agathobacter*, *Gemmiger*, *Streptococcus*, were negatively correlated with IFN-γ expression levels, whereas the opposite was observed for the genera with reduced abundance, such as *Phocaeicola* and *Parabacteroides*. In children with MDD, IL-17, a pro-inflammatory cytokine, was positively correlated with the genera displaying increased abundance and negatively correlated with those showing decreased abundance. Importantly, the beneficial butyrate-producing genus, *Faecalibacterium*, was negatively correlated with IL-17 expression. Our results suggested that changes in the cytokine profile of children with MDD were closely correlated with alterations in fecal microbiota abundance, and may be involved in the pathophysiology of MDD in childhood.

## Discussion

In our present study, we characterized the structure and composition of the fecal microbiota and host cytokine expression profile in drug-naïve Chinese school-aged children with MDD for the first time. In our cohort, we found changed overall structure of the fecal microbiota in children with MDD when compared with the healthy controls, specifically, unaltered bacterial α-diversity, increased richness indices such as ACE, Chao1, and observed OTUs and altered β-diversity. LEfSe identified several pro-inflammatory genera such as *Streptococcus* increased and anti-inflammatory genera such as *Faecalibacterium* decreased in pediatric MDD patients. These key differential functional genera, alone or in combination, could serve as novel and powerful non-invasive biomarkers to distinguish between pediatric MDD patients from healthy ones. Meanwhile, altered host cytokine expression profiles, increased levels of pro-inflammatory cytokines such as IL-17 and decreased levels of anti-inflammatory cytokines such as IFN-γ, were observed in pediatric MDD patients. Additionally, the pronounced correlation was detected between the differential cytokine abundance and changes in gut microbiota of children with MDD. These findings demonstrated gut dysbiosis concomitant with the activation of a complex pro-inflammatory response in pediatric MDD patients, which might be play vital roles in the pathogenesis of children depression.

As far as the bacterial diversity was concerned, our finding in children with MDD was inconsistent with previous case-control studies involving adolescent and adult patients with depression. Bacterial α-diversity is often used as a proxy for community stability and function, with increasing α-diversity values indicating a greater number of species, with more even representation, and/or greater biodiversity. In fact, there were no consensus on the changing patterns of the bacterial α-diversity among MDD-related clinical studies (25). In adult MDD patients, higher bacterial α-diversity as indicated by Shannon was observed in our previous study (10), while another two Chinese studies demonstrated lower Shannon index (26, 27). Similar to our present study on pediatric patients with MDD, five Chinese studies involving drug-naive adult patients with MDD from different regions showed that α-diversity indices, such as the Shannon and Simpson indices, did not differ significantly between patients and controls (11, 28–31). A comparative meta-analysis of α-diversity between adult patients with MDD and controls conducted by Sanada et al. also observed unaltered α-diversity in MDD patients (32). This might be associated with differences in the number of OTUs as well as in relative abundances (33). In addition, no significant differences were observed in bacterial α-diversity in studies investigating depression in adolescents (34) or attention-deficit/hyperactivity disorder in children (35). Regarding β-diversity in depression, studies have been relatively consistent in reporting differences in the overall community composition of the gut microbiota between patients and controls. In a systematic review of gut microbiota composition in MDD-related observational studies, differences in gut microbiota composition were reported in 87% of MDD β-diversity analyses (36). Similar patterns were found in several studies on depression in adults. In these latter studies, PCoA could separate patients with MDD and healthy controls into different clusters despite significant interpersonal variability (11, 26, 30, 37, 38). Jackson et al. reported that β-diversity distances were associated with symptoms of depression (39), while another study found no such association (40). Regarding richness, in our study, the greater number of OTUs was observed in child patients with MDD than in healthy children. These findings were consistent with those of our previous study on adult patients with MDD (10), but contrasted with the observed decrease in the number of species reported for adult patients with MDD in Irish and Chinese studies (26, 37). In Chinese children with autism spectrum disorder, higher microbial richness was also observed when compared with age- and BMI-matched normally developing children (41). Despite the reported inconsistencies, these bacterial diversity indices have been explored as non-invasive diagnostic biomarkers and treatment outcome predictors for depression. The discrepancy in MDD-associated gut microbiota diversity between children and adults might be due to the differences in baseline profiles of the gut microbiota (42–45). The gut microbiota of healthy children displays both functional and taxonomic differences with respect to those of adults and may also be more susceptible to environmental factors (46). Generally, the age-related baseline gut microbiota profile can be strongly influenced by intrinsic factors, the accumulation of environmental and dietary exposures, life-style, and intestinal maturation, among other factors (47). The existence of a strong correlation between gut microbiota diversity and health suggests that gut dysbiosis in childhood may contribute to the occurrence and development of MDD in children.

Although diversity-related findings have been inconsistent among age groups, specific bacterial taxa have been associated with MDD in studies that compared the gut microbiota of patients with that of controls. Overall, the fecal microbiota of children with MDD is characterized by a greater abundance of pro-inflammatory bacteria, such as *Escherichia*/*Shigella*, and a lower abundance of anti-inflammatory bacteria, such as *Bacteroides* and *Faecalibacterium*. *Bacteroides* (the core genus of the phylum Bacteroidetes) is one of the most abundant bacterial genera in the human colon, and members of this genus in the gut microbiota have been associated with health benefits, resistance to pathogens, as well as other host physiologic, metabolic, and immunologic phenotypes (48, 49). In agreement with our previous study on depression in adults, *Bacteroides* abundance was reported to be decreased in children with depression (10, 50); nevertheless, the specific contributions of *Bacteroides* to behavioral changes and the underlying mechanisms remain largely elusive. Interestingly, our data also demonstrated that *Bacteroides* genus was associated with systemic inflammation, suggesting that this genus may participate in modulating the host immunity in childhood depression (51). In fact, reports on the changing pattern of *Bacteroides* abundance and its association with depression have been inconsistent among studies. Overall, increasing evidence supported the beneficial roles of *Bacteroides* in regulating the development of childhood depression. Valles-Colomer et al. found that a lower relative abundance of *Bacteroides* in adults with depression was correlated with lower quality of life and higher prevalence of depression (52). Rhee et al. observed that the *Bacteroides* genus was negatively associated with the total HAMD score in adult patients with MDD (53). Strandwitz et al. demonstrated that the relative abundance of *Bacteroides* in feces was negatively correlated with brain signatures associated with depression (54). In fact, the functions of *Bacteroides* were significantly influenced by which *Bacteroides* species or strain is dominant in the gut (55). One study found that different *Bacteroides* species differentially modulate depression-like behavior *via* metabolism-mediated gut–brain signaling, especially through the tryptophan pathway (56). Specifically, recent studies found that colonization by *B. fragilis*, *B. uniformis*, and *B. caccae*, but not *B. ovatus*, recapitulated the negative effects of “depression microbiota” on behavior and neurogenesis, suggesting differential behavioral impacts of bacteria from the same genus. *B. ovatus*, a human gut commensal bacterium with anti-inflammatory properties and potential as a next-generation probiotic (57, 58), can reportedly influence the abundance of intestinal short-chain fatty acids (SCFAs) and neurotransmitters such as γ-aminobutyric acid (GABA) (59). *Bacteroides* spp. are the major bacterial producers of inhibitory neurotransmitter GABA in the human gut, and the reduced GABA levels have been associated with depressive-like behavior (60). The abundance of *Parabacteroides*, a genus in the phylum Bacteroidetes, was also found to be decreased in the fecal microbiota of children with MDD. *Parabacteroides* has been associated with positive health states, while its reduced abundance has been linked to negative effects on health. Preclinical studies have also found that *Parabacteroides* is negatively correlated with depressive behaviors but positively correlated with neurotransmitter metabolism (61, 62). Its regulatory roles in host metabolism, especially the production of succinate and secondary bile acids, might play a role in MDD in childhood (63).

One beneficial bacterium, *Faecalibacterium* (typical strain *F. prausnitzii*), has been proposed as a major factor in human intestinal health as well as a health biosensor (64). Interestingly, several studies have indicated that *Faecalibacterium* abundance is reduced in both children and adults with depression (32), as well as in several neuropsychiatric disorders such as multiple sclerosis, Alzheimer’s disease, and Parkinson’s disease (16, 17, 65). These observations suggest that *Faecalibacterium* plays a positive role in the modulation of the gut–brain axis. In adults with depression, *Faecalibacterium* abundance has been negatively correlated with the severity of depressive symptoms, as evidenced by HAMD and Montgomery–Asberg Depression Rating Scale (MADRS) scores (10). In the present study, we also identified a negative correlation between *Faecalibacterium* abundance and the levels of the pro-inflammatory cytokine IL-17 in children with MDD. *Faecalibacterium* is an acetate consumer and can produce butyrate and various bioactive anti-inflammatory molecules such as shikimic and salicylic acids (66). These bioactive metabolites can promote vagus nerve stimulation in the colon, microglia maturation and activation, and the production of brain-derived neurotrophic factor (BDNF), which might be directly involved in the pathogenesis of childhood depression *via* its effect on the gut–brain axis. Romano et al. observed that the butyrate-producing *Faecalibacterium* was consistently associated with improved quality-of-life indicators (65). Given its lower abundance in patients with depression, *Faecalibacterium* may serve as a biomarker for discriminating between individuals with MDD and healthy controls.

Additionally, the abundance of the genera *Prevotella* and *Klebsiella* was also found to be increased in Chinese pediatric patients with autism spectrum disorder and Chinese adult patients with MDD (67–69). Interestingly, Lin et al. reported that increased numbers of *Prevotella* and *Klebsiella* were significantly and positively correlated with the HAMD score (69), while the abundances of both genera were found to be reduced after successful MDD treatment (33, 69, 70). The authors proposed that these findings should be considered in the diagnosis and therapeutic monitoring of MDD in the future. Although members of the genus *Prevotella* are not normally considered to be pathogenic, the role of *Prevotella* in mucosal inflammation and the subsequent dissemination of pro-inflammatory mediators might be involved in the pathogenesis of MDD in childhood. *Klebsiella*, a genus of Gram-negative bacteria, can translocate lipopolysaccharides (LPS), thereby activating pro-inflammatory responses and inducing depressive-like behaviors. This effect may play a role in MDD pathophysiology in children. Interestingly, the abundance of the normally beneficial genus, *Bifidobacterium*, was found to be increased in child patients with MDD, which was in accordance with that reported for adults with this disorder (30, 33, 38, 71). These results may challenge the notion that *Bifidobacterium* always exerts beneficial effects on the host given that the functions of *Bifidobacterium* seem to be species- or strains-specific. Some species or strains of *Bifidobacterium* have been associated with lower scores on depression scales after regular consumption as probiotics (72). However, metagenomic-based studies have found that the relative abundance of two *Bifidobacterium* species—*B. longum* and *B. dentium*—is increased in adult patients with MDD (33, 38). Therefore, it seems that the predominant *Bifidobacterium* species or strain determines the effects (positive or negative) of this genus on depressive behavior. *Escherichia/Shigella* (family Enterobacteriaceae) are thought to be harmless; however, this group has been reported to be overrepresented in both children and adults with depression. In this study, we found that *Escherichia/Shigella* abundance was significantly and positively correlated with systemic inflammation, which may induce depressive symptoms (73). A greater abundance of *Escherichia/Shigella* could lead to the release of greater amounts of LPS into the plasma, resulting in increased blood–brain barrier permeability and chronic and persistent neuroinflammation, finally leading to depression (74). In addition, Chen et al. found that *Escherichia/Shigella* was positively associated with the severity of anxiety (75), while Cattaneo et al. proposed *Escherichia/Shigella* as a candidate pro-inflammatory taxon given that its abundance showed a positive correlation with the blood levels of IL-1β, CXCL2, and NLRP3 (76). As previously mentioned, gut dysbiosis, especially an increase in the numbers of pro-inflammatory bacteria and a decrease in those of anti-inflammatory bacteria, might contribute to the disruption of the intestinal mucosal barrier and the blood–brain barrier, leading to intestinal inflammation and neuroinflammation, a decrease in the concentrations of neurotransmitters and bacterial metabolites (e.g., SCFAs), and, consequently, childhood depression.

The criterium used for the diagnosis of depression in children is the same as that employed for the diagnosis of depression in adults, and is highly dependent on a wide variety of behavioral changes in the patients. In this study, the Firmicutes/Bacteroidetes ratio was significantly increased in pediatric patients with MDD relative to that in the controls, demonstrating that gut microbiota homeostasis was disrupted in the affected children. However, the Firmicutes/Bacteroidetes ratio in healthy gut naturally increases from 1 to 3 with growing age (43, 77), demonstrating the age-related dynamics of human microbiota composition. That’s why the increased Firmicutes/Bacteroidetes ratio in pediatric patients with MDD was not suitable for serving as biomarkers for clinical childhood depression diagnosis. With the deeply exploration of the gut–brain axis in children with depression, the novel potential non-invasive diagnostic tools, the aforementioned key functional gut bacteria, could be used to discriminate the pediatric patients with depression and healthy children. As in previous studies (16, 17, 23, 78), we found that several genera, such as *Streptococcus*, *Klebsiella*, *Gemmiger*, and *Escherichia/Shigella*, could serve as diagnostic factors for distinguishing between children with MDD and healthy controls. To improve the diagnostic performance, we employed multiple logistic regression analysis to identify the best combinations of the key functional genera (*Bacteroides*, *Streptococcus*, *Faecalibacterium*, *Dorea*, *Romboutsia*, and *Parabacteroides*) that could be used for the diagnosis of depression in childhood (AUC reached 0.987). This represents high diagnostic efficacy, and indicates the potential suitability of this identification method. In the future, it may be possible to diagnose depression in children based on changes in the abundance of microbial biomarkers. However, the diagnostic and therapeutic potential of microbiota-related biomarkers for childhood depression should still consider microbiota-associated confounders such as diet and hygiene.

This study had several limitations. First, because we only enrolled school-aged children, no direct comparison could be made between the gut microbiota of children and adults with depression. Second, this case-control discovery study only served to identify an association between the gut microbiota and depression in childhood, and no causal effects were explored (79). Longitudinal follow-up validation studies, microbiota-targeted interventional studies, and mechanistic studies using animal models should be undertaken to verify the causal effects of these key functional bacteria depression in children. Third, although we identified several clues linking gut microbiota-derived metabolites and childhood depression in this study, we did not investigate this association further. Additional metabolomics analysis is needed to provide direct evidence for a link between microbiota-associated metabolites and the development of depression in children.

In summary, school-aged children with depression displayed disrupted gut microbiota homeostasis when compared with healthy controls. The altered overall structure of the gut microbiota was characterized by increased richness index values (ACE, Chao 1, and observed species) and altered β-diversity. LEfSe analysis demonstrated that the abundance of several pro-inflammatory bacteria, such as *Prevotella*, *Bifidobacterium*, and *Escherichia*/*Shigella*, was increased, whereas that of anti-inflammatory bacteria, such as *Bacteroides* and *Faecalibacterium*, was decreased. The combination of *Bacteroides*, *Streptococcus*, *Faecalibacterium*, *Dorea*, *Romboutsia*, and *Parabacteroides* may serve as novel powerful biomarkers for distinguishing between children with depression and healthy children. We further found that the host cytokine profile was altered in child patients with depression, that is, the levels of pro-inflammatory cytokines were increased while those of anti-inflammatory cytokines were decreased. The close correlation identified between the altered fecal microbiota and systemic inflammation in the host suggested that the gut microbiota might potentially play a role in the pathophysiology of depression in childhood. Our findings provide novel insights into the pathogenesis of depression in school-aged children while key functional bacteria in the gut may serve as novel targets for the non-invasive diagnosis and patient-tailored early precise intervention in children with depression.

## Data availability statement

The data presented in the study are deposited in the GenBank Sequence Read Archive repository, accession number PRJNA846994.

## Ethics statement

These protocols for the study were approved by the Ethics Committee of Lishui Second People’s Hospital (Zhejiang, China) and written informed consent was obtained from their guardian before enrollment. Written informed consent to participate in this study was provided by the participants’ legal guardian/next of kin.

## Author contributions

ZL., LZ and QS conceived and designed the experiments. ZL, XY, FC, XL, YC, LS, GLJ, DZ, GZJ, HL, LZ and QS performed the experiments. ZL, LS and XL analyzed the data. ZL, LS and XL wrote the paper and edited the manuscript. All authors contributed to the article and approved the submitted version.

## Funding

This present work was funded by the grants of Key R&D Program of Zhejiang (2022C03060), Zhejiang Basic Public Welfare Research Project (LGF20H090016), the Nutrition and Care of Maternal and Child Research Fund Project of Guangzhou Biostime Institute of Nutrition and Care (2019BINCMCF045), the National Natural Science Foundation of China (81771724, 31700800, 81790631), the Research Project of Jinan Microecological Biomedicine Shandong Laboratory (JNL-2022033C), the Taishan Scholar Foundation of Shandong Province (tsqn202103119), the National S&T Major Project of China (2018YFC2000500), and the Foundation of China’s State Key Laboratory for Diagnosis and Treatment of Infectious Diseases.

## Acknowledgments

The authors thank all the participants who recruited patients in this study.

## Conflict of interest

The authors declare that they have no known competing financial interests or personal relationships that could have appeared to influence the work reported in this paper.

## Publisher’s note

All claims expressed in this article are solely those of the authors and do not necessarily represent those of their affiliated organizations, or those of the publisher, the editors and the reviewers. Any product that may be evaluated in this article, or claim that may be made by its manufacturer, is not guaranteed or endorsed by the publisher.

## Supplementary material

Supplementary Figure 1 Comparison of the ratio of Firmicutes/Bacteroidetes between childhood MDD patients and the healthy controls. *p < 0.05.
