# Supplementary figures and images for: Changes in fecal microbiota composition and the cytokine expression profile in school-aged children with depression: A case-control study
Source: Front Immunol. 2022 Aug 19;13:964910. doi: 10.3389/fimmu.2022.964910 (PMC9437487; doi:10.3389/fimmu.2022.964910)

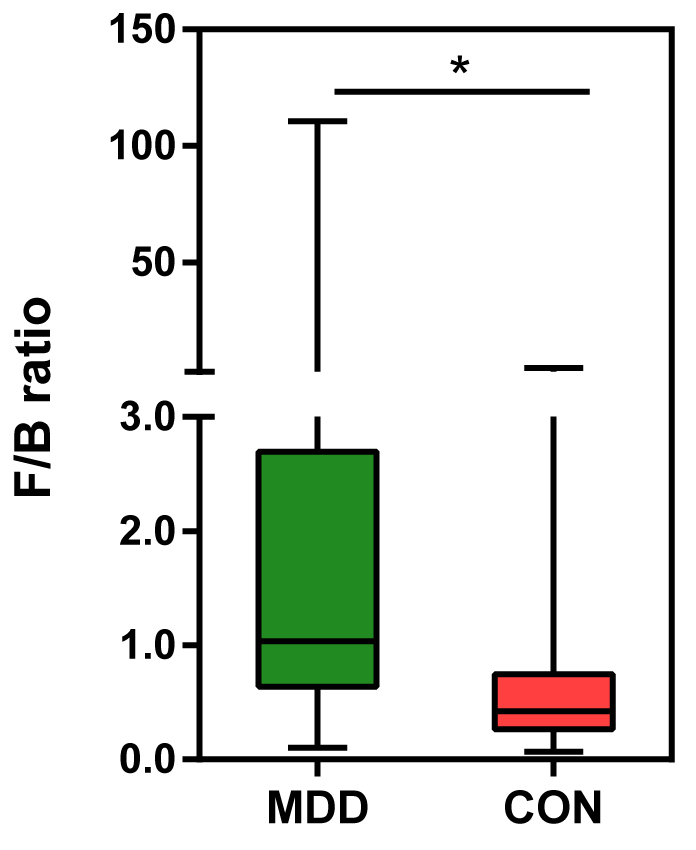

Supplement: Supplementary Figure 1 — Comparison of the ratio of Firmicutes/Bacteroidetes between childhood MDD patients and the healthy controls. *p < 0.05. [file Image_1.jpeg]
