# Supplementary material for: Changes in fecal microbiota composition and the cytokine expression profile in school-aged children with depression: A case-control study
Source: Front Immunol. 2022 Aug 19;13:964910. doi: 10.3389/fimmu.2022.964910 (PMC9437487; doi:10.3389/fimmu.2022.964910)
Supplement: Supplementary file 2 [file Table_1.docx]

**Table 1 Summary of the study subjects’ characteristics**

| **Characteristics** | **Control (n=48)** | **Patients (n=92)** |
| --- | --- | --- |
| Age (means ±SD, years) | 9.27±2.11 | 8.84±1.89 |
| Gender(Male/Female) | 22/26 | 42/50 |
| BMI(means ±SD) | 21.33±2.27 | 21.86±2.33 |
| Delivery mode |  |  |
| Vaginal, no | 37 | 68 |
| Cesarean section, no | 11 | 24 |
| Feeding mode |  |  |
| Vegetarian, no | 0 | 0 |
| Non-Vegetarian, no | 48 | 92 |
| Medical history |  |  |
| Active infections within 1 month, no | 0 | 0 |
| Psychiatric diseases, no | 0 | 0 |
| Autoimmune diseases, no | 0 | 0 |
| Antibiotics use within 1 month, no | 0 | 0 |
| Probiotics use within 1 month, no | 0 | 0 |
| Psychotropics within 1 month, no | 0 | 0 |
| Family history |  |  |
| Depression | 0 | 8 |
| Schizophrenia | 0 | 4 |
| HAMD | 4.2 ± 2.48 | 24.0 ± 4.52 |

*BMI: Body mass index; HAMD: Hamilton Depression Scale; no: number; SD, standard deviation.
